# *rac*-2,2′-Bipiperidine-1,1′-diium dibromide

**DOI:** 10.1107/S1600536811016084

**Published:** 2011-05-07

**Authors:** Marju Laars, Kerti Ausmees, Marina Kudrjashova, Tõnis Kanger, Franz Werner

**Affiliations:** a Tallinn University of Technology, Department of Chemistry, Akadeemia tee 15, 12618 Tallinn, Estonia

## Abstract

In the title compound, C_10_H_22_N_2_^2+^·2Br^−^, a precursor in the synthesis of organocatalysts, the bipiperidinium ion is located on a twofold rotation axis which passes through the mid-point of the central C—C bond. The piperidinium ring adopts a chair conformation. In the crystal, the cations are linked together by Br^−^ ions through N—H⋯Br hydrogen bonds, forming layers parallel to the *ab* plane.

## Related literature

For the synthesis, see: Krumholz (1953 ▶); Herrmann *et al.* (2006 ▶). For the application of *N*-substituted enanti­opure derivatives of the title compound in organocatalysis, see: Laars *et al.* (2008 ▶). For details of the Cu^II^–catalysed Henry reaction, see: Noole *et al.* (2010 ▶). For related structures, see: Sato *et al.* (1982 ▶); Baran *et al.* (1992*a* ▶,*b* ▶); Intini *et al.* (2008 ▶).
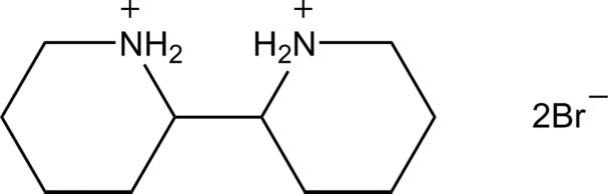


## Experimental

### 

#### Crystal data

C_10_H_22_N_2_^2+^·2Br^−^
*M* *_r_* = 330.12
Monoclinic, 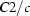

*a* = 11.789 (2) Å
*b* = 10.6403 (18) Å
*c* = 11.6632 (17) Å
β = 107.687 (5)°
*V* = 1393.9 (4) Å^3^
*Z* = 4
Mo *K*α radiation
μ = 5.79 mm^−1^
*T* = 300 K
0.40 × 0.30 × 0.20 mm

#### Data collection

Bruker SMART X2S diffractometer
Absorption correction: multi-scan (*SADABS*; Sheldrick, 1996 ▶) *T*_min_ = 0.151, *T*_max_ = 0.391
4225 measured reflections
1225 independent reflections
1012 reflections with *I* > 2σ(*I*)
*R*_int_ = 0.068

#### Refinement

*R*[*F* ^2^ > 2σ(*F* ^2^)] = 0.038
*wR*(*F* ^2^) = 0.093
*S* = 1.08
1224 reflections
70 parameters
H atoms treated by a mixture of independent and constrained refinement
Δρ_max_ = 0.47 e Å^−3^
Δρ_min_ = −0.94 e Å^−3^

### 

Data collection: *GIS* (Bruker, 2010 ▶); cell refinement: *SAINT* (Bruker, 2009 ▶); data reduction: *SAINT*; program(s) used to solve structure: *SHELXS97* (Sheldrick, 2008 ▶); program(s) used to refine structure: *SHELXL97* (Sheldrick, 2008 ▶); molecular graphics: *Mercury* (Macrae *et al.*, 2006 ▶); software used to prepare material for publication: *SHELXL97*.

## Supplementary Material

Crystal structure: contains datablocks global, I. DOI: 10.1107/S1600536811016084/is2700sup1.cif

Structure factors: contains datablocks I. DOI: 10.1107/S1600536811016084/is2700Isup2.hkl

Supplementary material file. DOI: 10.1107/S1600536811016084/is2700Isup3.cml

Additional supplementary materials:  crystallographic information; 3D view; checkCIF report

## Figures and Tables

**Table 1:** Hydrogen-bond geometry (Å, °)

*D*—H⋯*A*	*D*—H	H⋯*A*	*D*⋯*A*	*D*—H⋯*A*
N1—H1*NA*⋯Br1^i^	0.95 (4)	2.36 (4)	3.293 (3)	168 (3)
N1—H1*NB*⋯Br1^ii^	0.92 (4)	2.34 (4)	3.228 (3)	162 (3)

Symmetry codes: (i) 
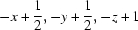
; (ii) 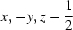.

**Fig. 1.**
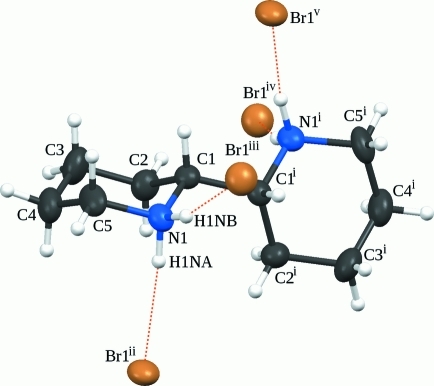
Cationic moiety in the crystal structure of the title compound together with the bromide ions bound to it through N—H···Br hydrogen bonds. Displacement ellipsoids for non–H atoms are drawn at the 50% probability level. Orange dashed lines indicate the hydrogen bonds. Symmetry codes: (i) -x, y, 1/2 - z; (ii) 1/2-x, 1/2 - y, 1 - z; (iii) x, -y, -1/2 + z; (iv) -1/2 + x, 1/2 - y, -1/2 + z; (v) -x, -y, 1 - z.

**Fig. 2.**
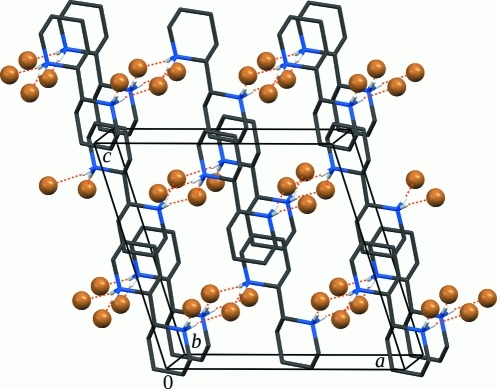
Packing diagram of the title compound. Orange dashed lines indicate N—H···Br hydrogen bonds. H atoms not involved in the hydrogen bonds have been omitted for clarity.

## supplementary crystallographic information

### Comment

*N*-substituted, enantiopure derivatives of the title phase, *rac*-2,2'-bipiperidine-1,1'-diium dibromide (I), catalyse stereoselectively both aldol reactions (Laars *et al.*, 2008) and, in the form of their Cu^II^–complexes, Henry (nitro-aldol) reactions (Noole *et al.*, 2010).

Owing to the twofold axis, passing the centre of the bond C1—C1^i^ (Fig. 1), *Z*'=0.5. Bond lengths and bond angles in the salt are normal. The piperidinium rings adopt chair conformation, with their least-squares planes (defined by their carbon and nitrogen atoms) twisted by about 77° against each other. Parallel to the (0 0 1) plane, the structure is made up of layers with a repeating distance of *d*_001_/2 of cations, which are hydrogen-bound *via* bromide ions (Fig. 2).

### Experimental

Single crystals of (I) were prepared from 2,2'-bipiperidine (Krumholz, 1953) according to Herrmann *et al.* (2006).

### Refinement

Except for the protonic H atoms H1NA and H1NB, whose positions were refined freely, H atoms were included at calculated positions [*d*(C—H) = 0.97 (CH_2_) or 0.98 Å (CH)] and treated as riding on their base atoms. For all H atoms, *U*_iso_(H) values were set at 1.2*U*_eq_(C or N). The 6 8 10 reflection was excluded from the refinement due to its large Δ(*F*^2^)/esd value.

### Crystal data

**Table:**

C_10_H_22_N_2_^2+^·2Br^−^	*F*(000) = 664
*M**_r_* = 330.12	*D*_x_ = 1.573 Mg m^−^^3^
Monoclinic, *C*2/*c*	Mo *K*α radiation, λ = 0.71073 Å
Hall symbol: -C 2yc	Cell parameters from 1621 reflections
*a* = 11.789 (2) Å	θ = 2.6–24.9°
*b* = 10.6403 (18) Å	µ = 5.79 mm^−^^1^
*c* = 11.6632 (17) Å	*T* = 300 K
β = 107.687 (5)°	Prism, colourless
*V* = 1393.9 (4) Å^3^	0.40 × 0.30 × 0.20 mm
*Z* = 4	

### Data collection

**Table:**

Bruker SMART X2S diffractometer	1225 independent reflections
Radiation source: XOS X-beam microfocus source	1012 reflections with *I* > 2σ(*I*)
doubly curved silicon crystal	*R*_int_ = 0.068
ω scans	θ_max_ = 25.0°, θ_min_ = 2.6°
Absorption correction: multi-scan (*SADABS*; Sheldrick, 1996)	*h* = −13→14
*T*_min_ = 0.151, *T*_max_ = 0.391	*k* = −12→12
4225 measured reflections	*l* = −13→13

### Refinement

**Table:**

Refinement on *F*^2^	Primary atom site location: structure-invariant direct methods
Least-squares matrix: full	Secondary atom site location: difference Fourier map
*R*[*F*^2^ > 2σ(*F*^2^)] = 0.038	Hydrogen site location: inferred from neighbouring sites
*wR*(*F*^2^) = 0.093	H atoms treated by a mixture of independent and constrained refinement
*S* = 1.08	*w* = 1/[σ^2^(*F*_o_^2^) + (0.*P*)^2^ + 0.0285*P*] where *P* = (*F*_o_^2^ + 2*F*_c_^2^)/3
1224 reflections	(Δ/σ)_max_ < 0.001
70 parameters	Δρ_max_ = 0.47 e Å^−^^3^
0 restraints	Δρ_min_ = −0.94 e Å^−^^3^

### Special details

**Table:**

Geometry. All e.s.d.'s (except the e.s.d. in the dihedral angle between two l.s. planes) are estimated using the full covariance matrix. The cell e.s.d.'s are taken into account individually in the estimation of e.s.d.'s in distances, angles and torsion angles; correlations between e.s.d.'s in cell parameters are only used when they are defined by crystal symmetry. An approximate (isotropic) treatment of cell e.s.d.'s is used for estimating e.s.d.'s involving l.s. planes.
Refinement. Refinement of *F*^2^ against ALL reflections. The weighted *R*-factor *wR* and goodness of fit *S* are based on *F*^2^, conventional *R*-factors *R* are based on *F*, with *F* set to zero for negative *F*^2^. The threshold expression of *F*^2^ > σ(*F*^2^) is used only for calculating *R*-factors(gt) *etc*. and is not relevant to the choice of reflections for refinement. *R*-factors based on *F*^2^ are statistically about twice as large as those based on *F*, and *R*- factors based on ALL data will be even larger.

### Fractional atomic coordinates and isotropic or equivalent isotropic displacement parameters (Å^2^)

**Table:**

	*x*	*y*	*z*	*U*_iso_*/*U*_eq_	
Br1	0.19514 (3)	0.07309 (4)	0.77393 (4)	0.0429 (2)	
N1	0.1066 (3)	0.1990 (3)	0.1590 (3)	0.0319 (7)	
H1NA	0.156 (3)	0.271 (4)	0.183 (3)	0.038*	
H1NB	0.146 (3)	0.132 (4)	0.202 (4)	0.038*	
C1	−0.0106 (3)	0.2219 (3)	0.1814 (3)	0.0287 (8)	
H1	−0.0630	0.1510	0.1473	0.034*	
C2	−0.0661 (3)	0.3394 (4)	0.1136 (3)	0.0385 (9)	
H2A	−0.1423	0.3553	0.1265	0.046*	
H2B	−0.0148	0.4111	0.1438	0.046*	
C3	−0.0836 (4)	0.3232 (5)	−0.0213 (4)	0.0524 (11)	
H3A	−0.1393	0.2553	−0.0526	0.063*	
H3B	−0.1169	0.3997	−0.0635	0.063*	
C4	0.0349 (4)	0.2940 (4)	−0.0433 (3)	0.0489 (11)	
H4A	0.0213	0.2782	−0.1284	0.059*	
H4B	0.0872	0.3662	−0.0208	0.059*	
C5	0.0944 (4)	0.1811 (4)	0.0280 (4)	0.0442 (10)	
H5A	0.0474	0.1064	−0.0017	0.053*	
H5B	0.1725	0.1694	0.0182	0.053*	

### Atomic displacement parameters (Å^2^)

**Table:**

	*U*^11^	*U*^22^	*U*^33^	*U*^12^	*U*^13^	*U*^23^
Br1	0.0502 (3)	0.0283 (3)	0.0495 (4)	−0.01100 (16)	0.0142 (3)	−0.00332 (18)
N1	0.0415 (17)	0.0201 (16)	0.0361 (19)	0.0024 (14)	0.0146 (16)	0.0033 (15)
C1	0.0344 (18)	0.0208 (18)	0.031 (2)	−0.0017 (15)	0.0101 (16)	−0.0031 (17)
C2	0.042 (2)	0.034 (2)	0.037 (2)	0.0072 (18)	0.0088 (19)	0.0033 (19)
C3	0.069 (3)	0.053 (3)	0.029 (2)	0.006 (2)	0.005 (2)	0.004 (2)
C4	0.075 (3)	0.044 (3)	0.031 (2)	−0.004 (2)	0.020 (2)	0.000 (2)
C5	0.068 (3)	0.033 (2)	0.040 (2)	−0.006 (2)	0.030 (2)	−0.012 (2)

### Geometric parameters (Å, °)

**Table:**

N1—C1	1.501 (4)	C2—H2A	0.9700
N1—C5	1.503 (5)	C3—C4	1.528 (5)
N1—H1NB	0.92 (4)	C3—H3A	0.9700
N1—H1NA	0.95 (4)	C3—H3B	0.9700
C1—C2	1.517 (5)	C4—C5	1.508 (6)
C1—C1^i^	1.542 (6)	C4—H4A	0.9700
C1—H1	0.9800	C4—H4B	0.9700
C2—C3	1.533 (5)	C5—H5A	0.9700
C2—H2B	0.9700	C5—H5B	0.9700
			
C1—N1—C5	112.9 (3)	C4—C3—C2	110.5 (3)
C1—N1—H1NB	112 (2)	C4—C3—H3A	109.5
C5—N1—H1NB	110 (2)	C2—C3—H3A	109.5
C1—N1—H1NA	109 (2)	C4—C3—H3B	109.5
C5—N1—H1NA	106 (2)	C2—C3—H3B	109.5
H1NB—N1—H1NA	107 (3)	H3A—C3—H3B	108.1
N1—C1—C2	108.5 (3)	C5—C4—C3	111.4 (3)
N1—C1—C1^i^	108.3 (3)	C5—C4—H4A	109.3
C2—C1—C1^i^	116.7 (2)	C3—C4—H4A	109.3
N1—C1—H1	107.7	C5—C4—H4B	109.3
C2—C1—H1	107.7	C3—C4—H4B	109.3
C1^i^—C1—H1	107.7	H4A—C4—H4B	108.0
C1—C2—C3	110.2 (3)	N1—C5—C4	110.2 (3)
C1—C2—H2B	109.6	N1—C5—H5A	109.6
C3—C2—H2B	109.6	C4—C5—H5A	109.6
C1—C2—H2A	109.6	N1—C5—H5B	109.6
C3—C2—H2A	109.6	C4—C5—H5B	109.6
H2B—C2—H2A	108.1	H5A—C5—H5B	108.1

Symmetry codes: (i) −*x*, *y*, −*z*+1/2.

### Hydrogen-bond geometry (Å, °)

**Table:**

*D*—H···*A*	*D*—H	H···*A*	*D*···*A*	*D*—H···*A*
N1—H1NA···Br1^ii^	0.95 (4)	2.36 (4)	3.293 (3)	168 (3)
N1—H1NB···Br1^iii^	0.92 (4)	2.34 (4)	3.228 (3)	162 (3)

Symmetry codes: (ii) −*x*+1/2, −*y*+1/2, −*z*+1; (iii) *x*, −*y*, *z*−1/2.
